# *In Silico* Prediction of Small Molecule-miRNA Associations Based on the HeteSim Algorithm

**DOI:** 10.1016/j.omtn.2018.12.002

**Published:** 2018-12-13

**Authors:** Jia Qu, Xing Chen, Ya-Zhou Sun, Yan Zhao, Shu-Bin Cai, Zhong Ming, Zhu-Hong You, Jian-Qiang Li

**Affiliations:** 1 School of Information and Control Engineering, China University of Mining and Technology, Xuzhou 221116, China; 2 College of Computer Science and Software Engineering, Shenzhen University, Shenzhen 518060, China; 3 Xinjiang Technical Institute of Physics and Chemistry, Chinese Academy of Science, Ürümqi 830011, China

**Keywords:** microRNA, small molecule, association prediction, HeteSim algorithm, heterogeneous network

## Abstract

Targeting microRNAs (miRNAs) with drug small molecules (SMs) is a new treatment method for many human complex diseases. Unsurprisingly, identification of potential miRNA-SM associations is helpful for pharmaceutical engineering and disease therapy in the field of medical research. In this paper, we developed a novel computational model of HeteSim-based inference for SM-miRNA Association prediction (HSSMMA) by implementing a path-based measurement method of HeteSim on a heterogeneous network combined with known miRNA-SM associations, integrated miRNA similarity, and integrated SM similarity. Through considering paths from an SM to a miRNA in the heterogeneous network, the model can capture the semantics information under each path and predict potential miRNA-SM associations based on all the considered paths. We performed global, miRNA-fixed local and SM-fixed local leave one out cross validation (LOOCV) as well as 5-fold cross validation based on the dataset of known miRNA-SM associations to evaluate the prediction performance of our approach. The results showed that HSSMMA gained the corresponding areas under the receiver operating characteristic (ROC) curve (AUCs) of 0.9913, 0.9902, 0.7989, and 0.9910 ± 0.0004 based on dataset 1 and AUCs of 0.7401, 0.8466, 0.6149, and 0.7451 ± 0.0054 based on dataset 2, respectively. In case studies, 2 of the top 10 and 13 of the top 50 predicted potential miRNA-SM associations were confirmed by published literature. We further implemented case studies to test whether HSSMMA was effective for new SMs without any known related miRNAs. The results from cross validation and case studies showed that HSSMMA could be a useful prediction tool for the identification of potential miRNA-SM associations.

## Introduction

Functional studies revealed that RNAs, once thought to be simple messengers from DNA to protein, have important roles in many cellular processes.^1^ They were found to regulate transcription, translation, RNA modification, mRNA stability, chromatin structure, and signaling pathways by interacting with various biological molecules.^2^ Most of these processes are related to a variety of human diseases, including cancers and neurodegenerative and neuromuscular diseases.^3^, ^4^ These discoveries have validated the potential of RNAs as therapeutic targets.^5^ Therefore, scientists have been excited about the prospect of the new class of drug targets, and they have developed novel tools to study the drug ability of RNAs.

For decades, small molecules (SMs) have been studied for their modulatory function by therapeutic targeting of proteins.^6^ While there are many examples of protein-targeted drug design, similar research for RNA targeting wasn’t uncovered until the late 1980s.^7^ Several classes of antibacterial drugs were found to be able to bind to bacterial rRNA to exert their function.^8^ To date, SMs have been found to target mammalian RNAs through various mechanisms. These RNAs are from various regions of the genome. They can be categorized into five general classes based on structure: RNA splicing, microRNAs (miRNAs), RNA repeat elements, G-quadruplex structures, and ribosomal synthesis.^9^

miRNAs are short 20- to 25-nt non-coding RNA (ncRNA) transcripts.^10^ They have important post-transcriptional cellular functions by binding to their target mRNA, resulting in mRNA decay or inhibition of translation.^11^ Many miRNAs are correlated with a variety of human diseases, including diabetes; obesity; cancers; and neurodegenerative, autoimmune, and cardiovascular diseases.^12^ Therefore, they are becoming potential SM drug targets.^13^ SMs that can bind to the Drosha- or Dicer-processing site target downstream miRNA to prevent the processing of mature miRNA formation or regulate function of miRNA. Several laboratories have reported SM inhibitors of Drosha- or Dicer-miRNA interactions. Disney et al.^14^ found that the molecule Targaprimir-96 could selectively target pri-miRNA 96 and, subsequently, inhibit Drosha processing in triple-negative breast cancer (TNBC). In a separate report, they found that ligand Targapremir-210 targeted pre-miRNA-210 and inhibited Dicer processing under hypoxic conditions.^15^ Another search yielded a ligand bis-benzimidazole to target miRNA-544; by targeting a functional miRNA-processing site, it achieved successful miRNA-544 inhibition.^16^

To identify SM-RNA associations, several high-throughput-screening approaches have been developed. They are based on mass spectrometry, fluorescence, or reporter.^17^, ^18^, ^19^, ^20^ Currently, reporter-based assays are most commonly used in the identification of miRNA-targeted SM inhibitors.^12^ The principle of this method is that the luciferase activity can be suppressed when miRNA binds to the 3′ UTR sequence of its target luciferase gene. If SM can target miRNA and decrease the binding, the luciferase readout of the labeled mRNA will increase.^12^ However, this assay could not provide information about specific miRNA-gene interaction or direct miRNA engagement that the SM may inhibit.

On the other hand, several informatics-based high-throughput-screening methods that measure direct miRNA binding have been developed to aid miRNA-targeted drug discovery. For example, through two-dimensional combinatorial screening (2DCS)^21^ and statistical method analyzing structure-activity relationships based on sequence (StARTS),^22^ Disney et al.^14^ constructed the RNA motif-SM database named Inforna. These approaches have aided in the discovery of miRNA-SM association, though the further mechanisms of association were revealed during follow-up biological experiments rather than during the screening. Recently, Lorenz and Garner^23^ reported a novel high-throughput-screening approach that may compensate for these drawbacks. This approach can detect direct inhibitors of miRNA cleavage, and it may thus be promising for discovering SMs that target specific pre-miRNA.

The research of miRNA-SM associations has significantly advanced over the past few years. Continued development of both fundamental mechanisms and biological approaches has further benefited the discovery of miRNA-targeted SM ligands. However, the identification and validation of miRNA-SM associations by biological experiment are still always time consuming and costly. It is urgent for the scientists to elucidate how to select the best-suited miRNAs for targeting by more efficient and accurate methods. Developing computational prediction models for miRNA-SM associations is a promising strategy. The efficient and reliable prediction models can lead to the efficient design of miRNA-targeted ligands. Predicting miRNA-SM association will also be crucial for the selectivity of SM targeting miRNA and establishing guidelines for SM targeting miRNA research. This work would significantly aid in our understanding of the relationships between SMs and miRNAs. Also, on the basis of the association information between miRNAs and SMs, we could further predict synergistic drug combinations.^24^ Therefore, there is an urgent need to develop new computational approaches or models to speed up the studies of this field.

In recent years, a number of studies based on computational models have been developed on the identification of potential miRNA-SM associations, which may be helpful to a new direction of miRNA-targeted therapies.^12^, ^25^, ^26^, ^27^ Wang et al.^28^ introduced a new method to predict potential miRNA-SM associations through calculating a functional similarity score of each miRNA-SM pair. In the model, they identified differentially expressed genes for drug treatment and miRNA perturbation, and then they calculated the functional similarity of each miRNA-SM pair by using gene ontology (GO) enrichment analysis on their differentially expressed genes. Meng et al.^29^ proposed a computational approach to identify the potential associations between SMs and Alzheimer’s disease (AD)-related miRNAs based on the differentially expressed target genes that were regulated by the aberrantly expressed AD-related miRNAs. Differentially expressed target genes used in the model were divided into two groups based on whether they were overexpressed or under-expressed, and Kolmogorov-Smirnov (KS) values for the overexpressed and under-expressed target genes were calculated respectively to obtain final scores for the potential associations between SMs and AD-related miRNAs by integrating the two KS values. Besides, Jiang et al.^30^ have done the similar work for the identification of potential miRNA-SM associations in 23 different cancers through implementing a KS test based on the differential expression of miRNA target genes and transcriptional responses following drug treatment. They also constructed the SM-miRNA network for each cancer to analyze the property of each association, which could be helpful to the identification of drug candidates for cancer therapy.

Lv et al.^31^ developed a network-based computational method to predict the potential associations between miRNAs and SMs by implementing random walk with restart algorithm (RWR) on the integrated network that is composed of known miRNA-SM associations and SM-SM and miRNA-miRNA similarity. In addition, Li et al.^32^ also proposed a network-based framework named predictive SM-miRNA Network-Based Inference (SMiR-NBI), which established a network by connecting drugs, miRNAs, and genes and implemented a network-based inference (NBI) framework on the network to prioritize miRNAs for SMs. Recently, Qu et al.^33^ put forward a calculation approach of Triple Layer Heterogeneous Network based SM-miRNA Association prediction (TLHNSMMA), in which they collected heterogeneous data about SMs, miRNAs, and diseases and treated disease information as a bridge to build a three-layer mixing network. On this basis, two iterative updating processes that spread information of heterogeneous data were generated to infer novel miRNA-SM associations and miRNA-disease associations simultaneously.

In this paper, drawing on previous research on the development of computational models on the miRNA-disease association prediction,^34^, ^35^, ^36^, ^37^, ^38^ we introduced a novel computational model of HeteSim-based inference for SM-miRNA Association prediction (HSSMMA) to calculate the relevance between miRNAs and SMs by implementing a path-based measurement method of HeteSim in a heterogeneous network that was constructed based on integrated miRNA similarity, integrated SM similarity, and experimentally confirmed miRNA-SM associations. The HeteSim is a path-constrained measurement method to calculate the relatedness of objects with the same or different types in a heterogeneous network based on the search path that connect two objects by a sequence of node types.^39^ In addition, the dataset of experimentally confirmed miRNA-SM associations used in this model was downloaded from the database of SM2miR.^40^ According to the known miRNA-SM associations, we constructed two types of datasets (dataset 1 and dataset 2), and we implemented HSSMMA on them for the identification of potential miRNA-SM associations, respectively. In dataset 1, only a part of SMs and miRNAs are involved in the known miRNA-SM associations. In dataset 2, all SMs and miRNAs are involved in the known miRNA-SM associations.

To test the prediction performance of HSSMMA, we employed global, miRNA-fixed local and SM-fixed local leave one out cross validation (LOOCV) as well as 5-fold cross validation on the two datasets, respectively. The results showed that the area under the receiver operating characteristic (ROC) curve (AUC) of global LOOCV was 0.9913 and 0.7401 based on dataset 1 and dataset 2, respectively. Through fixing each miRNA to predict miRNA-associated SMs, the AUC of local LOOCV was, respectively, 0.9902 and 0.8466 for the dataset 1 and dataset 2. Through fixing each SM to predict SM-associated miRNAs, the AUC of local LOOCV was, respectively, 0.7989 and 0.6149 for the dataset 1 and dataset 2. For 5-fold cross validation, the average AUC and corresponding SD was 0.9910 ± 0.0004 and 0.7451 ± 0.0054 for the dataset 1 and dataset 2, respectively. In case studies, the results showed that 2 of the top 10 and 13 of the top 50 predicted miRNA-SM associations were confirmed by published references. We further implemented our model to the SMs without any known associated miRNAs. The known related miRNAs for the investigated SM would be removed from the training dataset and 5-fluorouracil, 17β-estradiol, and 5-aza-2′-deoxycytidine were taken as the investigated SMs, respectively. We found that 27, 24, and 26 of the top 50 predicted miRNAs for 5-fluorouracil (5-FU), 17β-estradiol (E2), and 5-aza-2′-deoxycytidine (5-Aza-CdR) were confirmed by SM2miR database and published literature reports, respectively.

## Results

### Performance Evaluation

For evaluating the prediction performance of HSSMMA, global, miRNA-fixed local, and SM-fixed local LOOCV as well as 5-fold cross validation were implemented based on the dataset of known miRNA-SM associations. We further compared the performance of HSSMMA with one previous classical computational model of SMiR-NBI based on dataset 1 and dataset 2, respectively. In LOOCV, we regarded each known miRNA-SM pair as a test sample in turn; the remaining known associations between miRNAs and SMs were considered as training samples. The miRNA-SM pairs without known associations were used as candidate samples. We would obtain the prediction scores of each miRNA-SM pair after HSSMMA was implemented. In the global LOOCV evaluation, we would compare the score of the test sample with the scores of all the candidate samples. In the miRNA-fixed local LOOCV, we would sort the score of the test sample with the scores of candidate samples that were made up of the pairs between SMs and fixed miRNAs. Meanwhile, in the SM-fixed local LOOCV, we would sort the score of the test sample with the scores of candidate samples that were made up of the all pairs between miRNAs and fixed SMs.

In 5-fold cross validation, we would randomly divide the known miRNA-SM associations into five equal parts; one part was selected as the test sample in turn and the rest (four parts) were regarded as training samples. In the same way, the miRNA-SM pairs without any known associations would be considered as candidate samples and the score of each test sample would be ranked with all candidate samples, respectively. Because of the random partition of the original samples in 5-fold cross validation, the corresponding validation results would be different in each random process. Therefore, we repeated the process of 5-fold cross validation 100 times in this study, and we took the average of the 100 cross validation results to evaluate the model.

**Figure 1:**
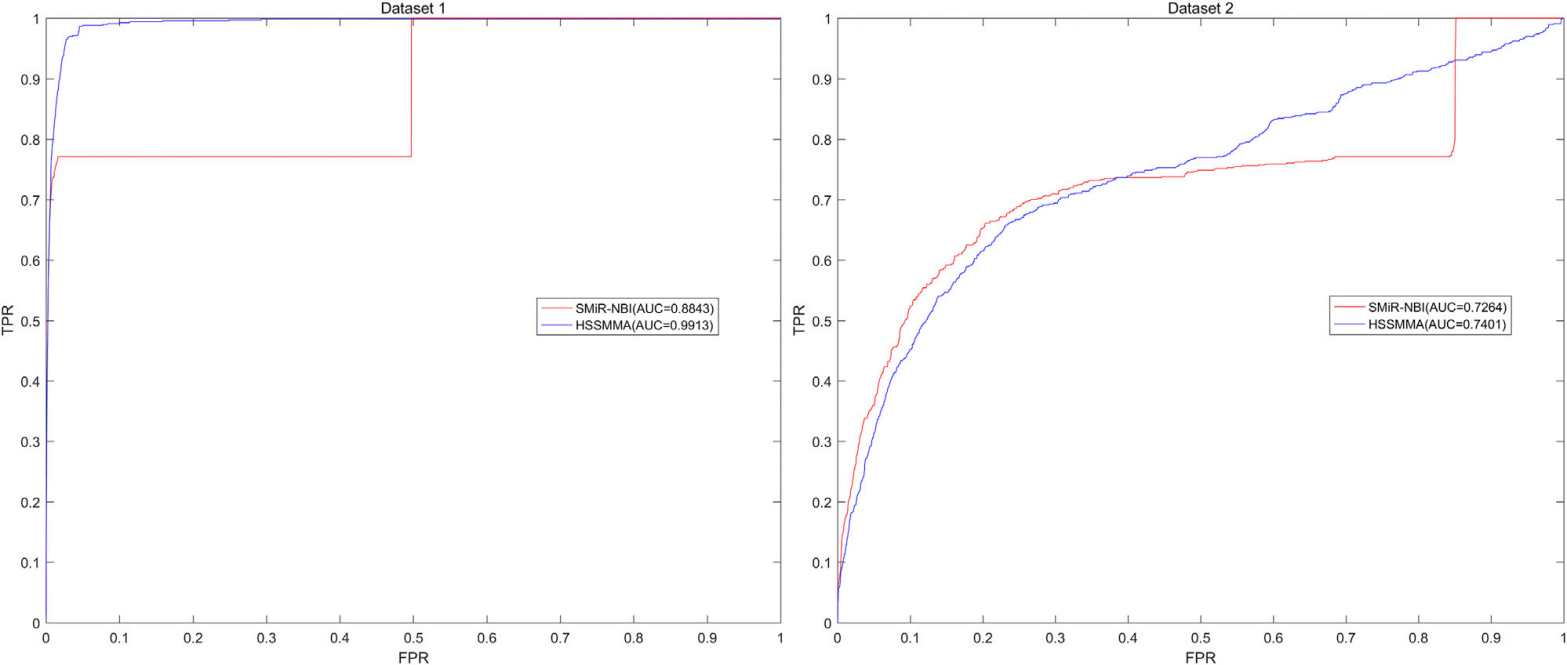
Performance Comparison between HSSMMA and SMiR-NBI in Terms of ROC Curves and AUCs Based on Global LOOCV HSSMMA obtained AUCs of 0.9913 and 0.7401 based on dataset 1 and dataset 2, respectively.

**Figure 2:**
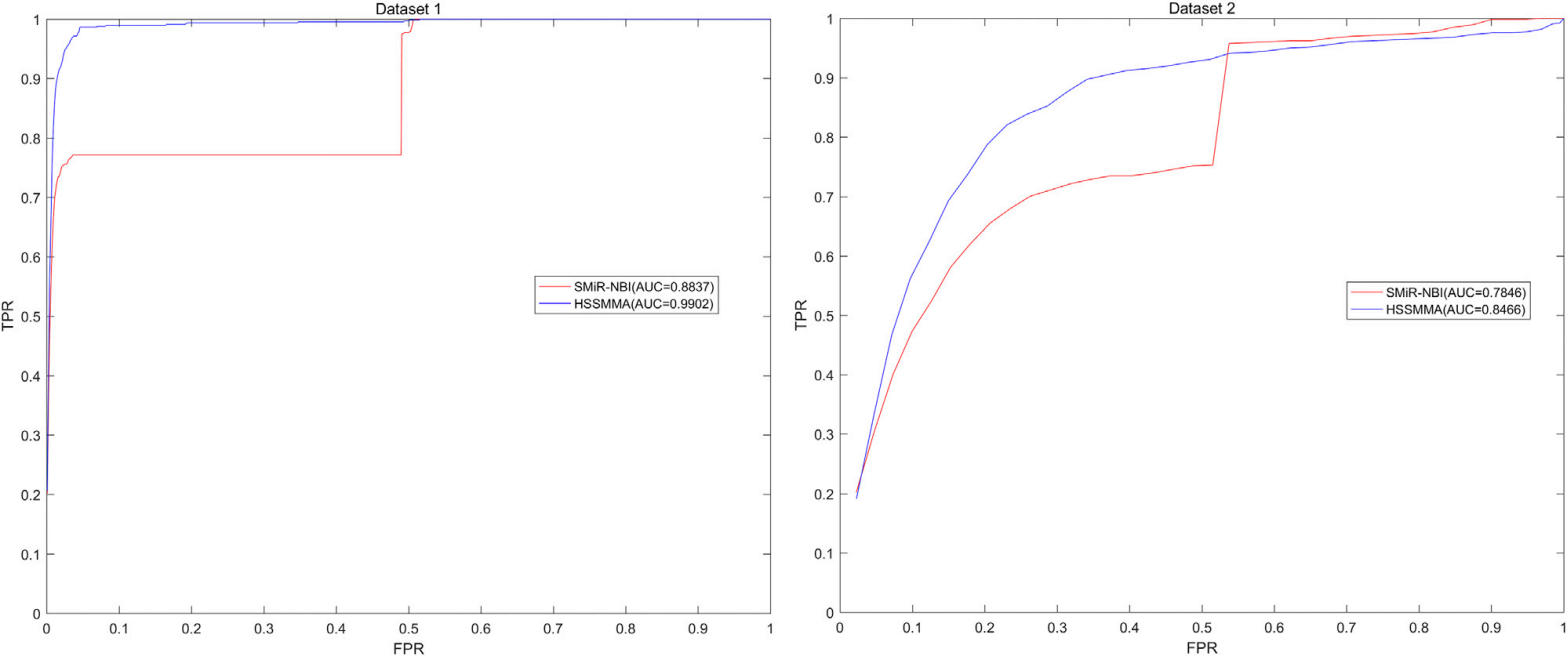
Performance Comparison between HSSMMA and SMiR-NBI in Terms of ROC Curves and AUCs Based on Local LOOCV by Fixing miRNAs to Rank SMs HSSMMA obtained AUCs of 0.9902 and 0.8466 based on dataset 1 and dataset 2, respectively.

**Figure 3:**
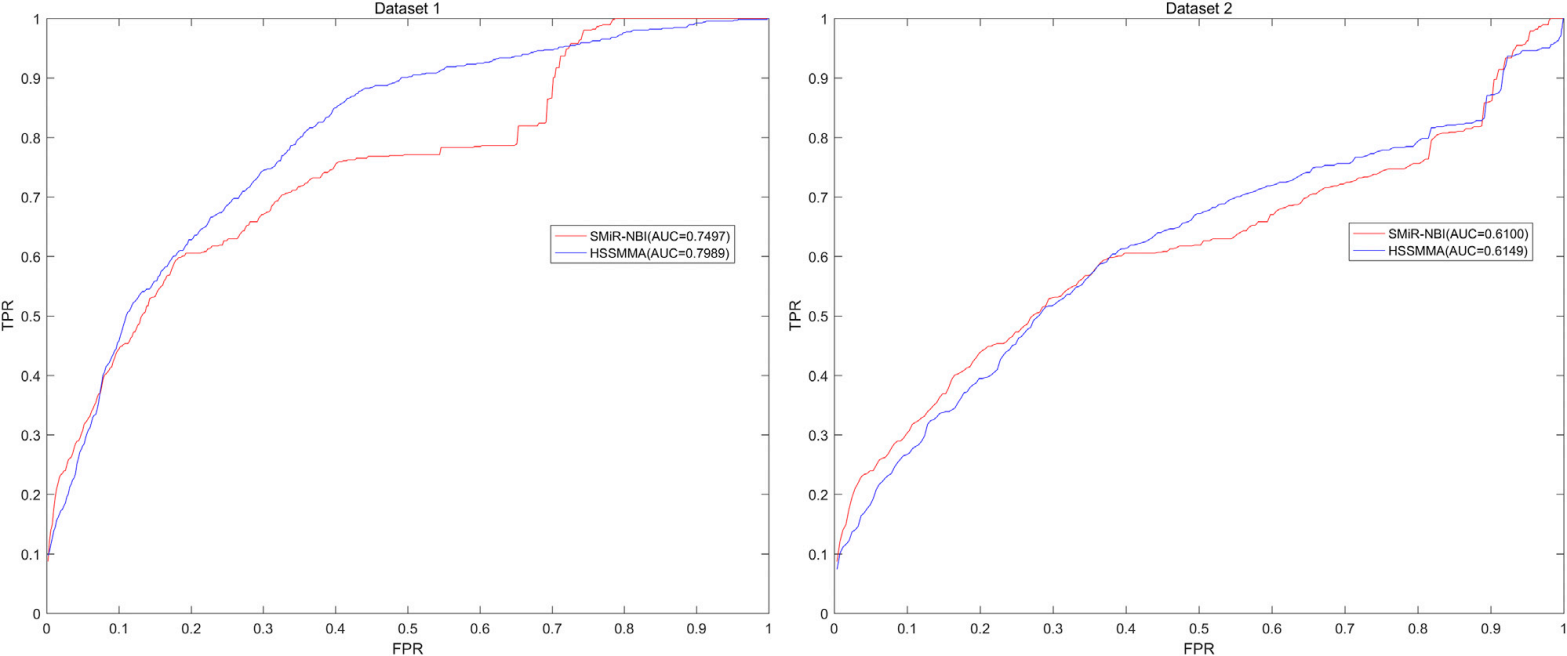
Performance Comparison between HSSMMA and SMiR-NBI in Terms of ROC Curves and AUCs Based on Local LOOCV by Fixing SMs to Rank miRNAs HSSMMA obtained AUCs of 0.7989 and 0.6149 based on dataset 1 and dataset 2, respectively.

At last, we plotted the ROC curve using true positive rate (TPR, sensitivity) against the false positive rate (FPR, 1-specificity) at different thresholds. Sensitivity denotes the percentage of test samples that were identified with higher ranks than the given threshold. Specificity refers to the percentage of negative miRNA-SM pairs with lower ranks than the threshold. After that, we calculated AUC as an evaluation index for the prediction performance of HSSMMA. If the value of AUC is 0.5, the prediction performance of the HSSMMA is random; if the value of AUC is 1, the prediction performance of HSSMMA is perfect. In the global LOOCV, the results showed that HSSMMA and SMiR-NBI obtained AUCs of 0.9913 and 0.8843 based on dataset 1, respectively, and AUCs of 0.7401 and 0.7264 based on dataset 2, respectively (see Figure 1). In the framework of miRNA-fixed local LOOCV, HSSMMA and SMiR-NBI obtained AUCs of 0.9902 and 0.8837, respectively, based on dataset 1 and AUCs of 0.8466 and 0.7846, respectively, based on dataset 2 (see Figure 2). In the framework of SM-fixed local LOOCV, HSSMMA and SMiR-NBI obtained AUCs of 0.7989 and 0.7497, respectively, based on dataset 1 and AUCs of 0.6149 and 0.6100, respectively, based on dataset 2 (see Figure 3). In 5-fold cross validation, HSSMMA and SMiR-NBI obtained AUCs of 0.9910 ± 0.0004 and 0.8554 ± 0.0063, respectively, based on dataset 1 and AUCs of 0.7451 ± 0.0054 and 0.7104 ± 0.0087, respectively, based on dataset 2. Comparisons between evaluation results of HSSMMA and SMiR-NBI demonstrate that HSSMMA is reliable and effective for the identification of potential miRNA-SM associations.

### Case Studies

We carried out a case study based on the dataset 1 to evaluate the effectiveness of the HSSMMA. After the implementation of HSSMMA, we observed the number of the verified miRNA-SM associations in the top 10, top 20, and top 50 prediction list. As the results showed, among the top 10, 20, and 50 potential SM-miRNA associations, there were 2, 4, and 13 associations confirmed by experiments, respectively (see Table 1). It is worth noting that the SM was presented using PubChem compound identifier (PubChem-CID) in the dataset of known miRNA-SM associations.

**Table 1:** Prediction of the Top 50 Potential miRNA-Related SMs Based on Dataset 1

SM	miRNA	Evidence	SM	miRNA	Evidence
CID 451668	hsa-mir-203a	26577858	CID 9444	hsa-mir-21	unconfirmed
CID 451668	hsa-mir-23a	unconfirmed	CID 451668	hsa-let-7b	26708866
CID 451668	hsa-mir-15b	unconfirmed	CID 451668	hsa-let-7e	22053057
CID 451668	hsa-let-7a-1	unconfirmed	CID 451668	hsa-mir-199a-1	unconfirmed
CID 60750	hsa-mir-23a	unconfirmed	CID 451668	hsa-mir-152	unconfirmed
CID 9444	hsa-mir-106b	unconfirmed	CID 3385	hsa-mir-181a-1	unconfirmed
CID 451668	hsa-let-7d	26802971	CID 451668	hsa-mir-146a	24885368
CID 451668	hsa-mir-132	unconfirmed	CID 3385	hsa-let-7b	25789066
CID 9444	hsa-mir-93	unconfirmed	CID 36462	hsa-let-7f-1	unconfirmed
CID 9444	hsa-mir-16-1	unconfirmed	CID 5288826	hsa-mir-17	unconfirmed
CID 451668	hsa-mir-128-2	unconfirmed	CID 9444	hsa-let-7f-1	unconfirmed
CID 9444	hsa-mir-16-2	unconfirmed	CID 3229	hsa-let-7g	unconfirmed
CID 451668	hsa-mir-18a	unconfirmed	CID 60750	hsa-mir-17	23001407
CID 9444	hsa-mir-210	unconfirmed	CID 3229	hsa-let-7e	unconfirmed
CID 451668	hsa-mir-128-1	27705931	CID 9444	hsa-mir-191	unconfirmed
CID 9444	hsa-mir-15a	unconfirmed	CID 60750	hsa-mir-24-2	25841339
CID 451668	hsa-let-7a-2	unconfirmed	CID 451668	hsa-mir-30e	unconfirmed
CID 451668	hsa-mir-199a-2	unconfirmed	CID 451668	hsa-mir-26b	unconfirmed
CID 451668	hsa-mir-92a-1	unconfirmed	CID 3385	hsa-mir-26a-1	unconfirmed
CID 451668	hsa-let-7a-3	26227220	CID 3385	hsa-mir-155	28347920
CID 9444	hsa-mir-25	unconfirmed	CID 451668	hsa-mir-342	unconfirmed
CID 451668	hsa-mir-106a	unconfirmed	CID 5288826	hsa-let-7f-1	unconfirmed
CID 3229	hsa-mir-24-1	unconfirmed	CID 9444	hsa-mir-23b	unconfirmed
CID 451668	hsa-mir-26a-1	unconfirmed	CID 451668	hsa-mir-200b	23626803
CID 3385	hsa-mir-126	26062749	CID 36462	hsa-mir-23b	unconfirmed

The SM was presented using the PubChem compound identifier (CID) in the dataset of known miRNA-SM associations. The first two columns record the top 1–25 miRNA-SM associations. The fourth and fifth columns record the top 26–50 miRNA-SM associations. The evidence for the associations was recent experimental literature with the corresponding PubMed ID.

For instance, in the top 10 predicted miRNA-SM associations, the predicted association between mir-203a and 5-Aza-CdR is ranked first. Recent study revealed that treatment of esophageal squamous cell carcinoma (ESCC) cells with 5-Aza-CdR resulted in increased miR-203a expression.^41^ The seventh predicted association is between let-7d and 5-Aza-CdR. Zhang et al.^42^ found that let-7d could inhibit dopamine D3 receptor (DRD3) expression in immortalized renal proximal tubule (RPT) cells via methylation. This inhibition could be abolished by 5-Aza-CdR. In the top 20 predicted miRNA-SM associations, we also revealed the potential association between miR-128-1 and 5-Aza-CdR ranked 15th. Shan et al.^43^ found that miR128-1 is downregulated and closely associated with glioblastoma multiforme (GBM). Treatment with the 5-Aza-CdR resulted in miR128-1 upregulation in GBM cells, and it inhibited tumor cell proliferation, suggesting that 5-Aza-CdR may potentially treat GBM by upregulating miR-128-1.^43^ Besides, the 20th predicted association between let-7a-3 and 5-Aza-CdR was verified. Zhu et al.^44^ found that treatment with 5-Aza-CdR could decrease the methylation density of let-7a-3 promoter and increase the level of let-7a-3 expression in acute myeloid leukemia (AML) cells. We further used HSSMMA to predict candidate miRNAs for all SMs in dataset 1, and the corresponding probability scores are provided (see Table S1).

To validate the prediction effectiveness for new SMs without any known related miRNAs, we carried out another case study based on dataset 1 by removing all associations between miRNAs and the investigated SM and implementing HSSMMA based on the rest of the known miRNA-SM associations. Then all predicted miRNAs for the investigated SM would be ranked according to their predicted scores, and the top 50 potential associations between miRNAs and the investigated SM would be confirmed by the SM2miR database and published references. Here, 5-FU, E2, and 5-Aza-CdR were taken as the investigated SMs, respectively.

### 5-FU

The agent 5-FU is a widely used chemotherapeutic drug in cancers.^45^ It induces cytotoxic effects by altering DNA and RNA metabolism and mRNA expression.^46^, ^47^, ^48^ Exposure to 5-FU promotes transcriptional reprogramming, leading to the alteration of mRNA or miRNA expression profiles that results in a change of cell fate.^49^, ^50^, ^51^ After implementing HSSMMA, we got the total ranking of potential miRNAs associated with 5-FU. As the results show, among the top 10 potential 5-FU-related miRNAs, there are 7 confirmed miRNAs, among which 6 miRNAs were confirmed by the known SM-miRNA association dataset constructed in the literature^31^ and 1 miRNA was confirmed by experimental report from the literature.^52^ Among the top 50 potential 5-FU-related miRNAs, there are 27 confirmed miRNAs, among which 19 miRNAs were confirmed by the known SM-miRNA association dataset constructed in the literature^31^ and 8 miRNAs were confirmed by experimental report from the literature (see Table 2).

**Table 2:** Top 50 miRNAs Associated with 5-Fluorouracil Were Predicted by HSSMMA Based on Dataset 1

SM	miRNA	Evidence	SM	miRNA	Evidence
CID 3385	hsa-mir-324	unconfirmed	CID 3385	hsa-mir-155	28347920
CID 3385	hsa-mir-24-1	26198104	CID 3385	hsa-mir-320a	26198104
CID 3385	hsa-mir-23a	26198104	CID 3385	hsa-mir-126	26062749
CID 3385	hsa-mir-24-2	26198104	CID 3385	hsa-mir-1226	26198104
CID 3385	hsa-mir-500a	unconfirmed	CID 3385	hsa-mir-409	unconfirmed
CID 3385	hsa-mir-501	26198104	CID 3385	hsa-mir-197	26198104
CID 3385	hsa-mir-181a-1	unconfirmed	CID 3385	hsa-mir-27b	26198104
CID 3385	hsa-mir-21	26198104	CID 3385	hsa-mir-345	unconfirmed
CID 3385	hsa-mir-27a	26198104	CID 3385	hsa-mir-132	26198104
CID 3385	hsa-mir-181a-2	24462870	CID 3385	hsa-let-7d	26198104
CID 3385	hsa-let-7b	25789066	CID 3385	hsa-mir-199a-2	26198104
CID 3385	hsa-mir-874	27221209	CID 3385	hsa-mir-128-2	26198104
CID 3385	hsa-mir-16-1	26198104	CID 3385	hsa-mir-299	unconfirmed
CID 3385	hsa-let-7a-1	26198104	CID 3385	hsa-mir-205	24396484
CID 3385	hsa-mir-650	unconfirmed	CID 3385	hsa-mir-373	–
CID 3385	hsa-mir-125b-1	unconfirmed	CID 3385	hsa-mir-128-1	26198104
CID 3385	hsa-mir-26a-1	unconfirmed	CID 3385	hsa-mir-342	26198104
CID 3385	hsa-mir-125b-2	unconfirmed	CID 3385	hsa-mir-194-1	unconfirmed
CID 3385	hsa-mir-124-1	unconfirmed	CID 3385	hsa-let-7c	25951903
CID 3385	hsa-mir-181b-1	unconfirmed	CID 3385	hsa-mir-149	26198104
CID 3385	hsa-mir-328	unconfirmed	CID 3385	hsa-mir-186	unconfirmed
CID 3385	hsa-mir-124-2	unconfirmed	CID 3385	hsa-mir-154	unconfirmed
CID 3385	hsa-mir-124-3	unconfirmed	CID 3385	hsa-mir-204	27095441
CID 3385	hsa-mir-346	unconfirmed	CID 3385	hsa-mir-337	unconfirmed
CID 3385	hsa-mir-181b-2	unconfirmed	CID 3385	hsa-mir-1-2	28347920

The top 1–25 miRNAs are shown in the second column while the top 26–50 are in the fifth column. As a result, 7 and 27 of the top 10 and top 50 were confirmed by the known experimental literature, respectively.

For instance, Huang et al.^52^ found that knockdown of astrocyte-elevated gene-1 (AEG-1) in colorectal cancer (CRC) cells can improve the expression of miR-181a-2 and increase the sensitivity of CRC cells to 5-FU, suggesting a potential mechanism to improve the efficiency of 5-FU by miR-181a-2. The association between let-7b and 5-FU was confirmed by Wang et al.^53^ They found that let-7b can decrease the expression of B cell lymphoma-extra large (Bcl-xL) and sensitize breast cancer cells to 5-FU.^53^ Through functional assays, Han et al.^54^ revealed that restoration of miR-874 could inhibit the proliferation of the CRC cells and decrease the 5-FU resistance of the CRC cells. In TNBC, the expression level of miR-205 was significantly elevated in MDA-MB-453 LAR-type TNBC tumor cells treated with 5-FU together with ixabepilone, suggesting the drugs may exert effects through the regulation of miR-205.^55^

### E2

The small molecular E2 is a type of human estrogen that exerts important effects on the reproductive and other related biological processes in both men and women.^56^ It has significant anticancer activity against human breast cancer.^57^ After implementing HSSMMA, we got the total ranking of miRNAs for E2. In the top 10 miRNAs, 5 miRNAs were confirmed by the database of SM2miR^31^ and 1 miRNA was confirmed by previous literature.^58^ Moreover, 14 of the top 50 miRNAs were verified by SM2miR and 10 were confirmed by experimental literature (see Table 3). For example, Aqil et al.^58^ confirmed that the overexpression of miR-375 following E2 treatment could be significantly protected by jamun diet. Li et al.^59^ demonstrated that miR-22 could effectively reverse E2-induced cell proliferation and invasion of tumor cells in endometrial endometrioid carcinomas (EECs) by inhibiting Cyclin D1 expression and the secretion of matrix metalloproteinase (MMP)-2 and MMP-9. Zhang et al.^60^ reported that miR-320a expression is significantly downregulated in cumulus granulosa cells (CCs) from polycystic ovary syndrome (PCOS) patients and this downregulation promotes E2 deficiency in CCs. Treatment of E2-sensitive MCF7 breast cancer cells with fulvestrant resulted in increased expression of endogenous miR-221.^61^

**Table 3:** Top 50 miRNAs Associated with 17β-Estradiol Were Predicted by HSSMMA Based on Dataset 1

SM	miRNA	Evidence	SM	miRNA	Evidence
CID 5757	hsa-mir-375	27030099	CID 5757	hsa-mir-146b	29331043
CID 5757	hsa-mir-324	unconfirmed	CID 5757	hsa-mir-17	26198104
CID 5757	hsa-mir-23a	26198104	CID 5757	hsa-mir-125b-1	unconfirmed
CID 5757	hsa-mir-21	26198104	CID 5757	hsa-mir-345	unconfirmed
CID 5757	hsa-mir-194-1	unconfirmed	CID 5757	hsa-mir-125b-2	unconfirmed
CID 5757	hsa-mir-24-1	unconfirmed	CID 5757	hsa-mir-195	unconfirmed
CID 5757	hsa-mir-124-1	26198104	CID 5757	hsa-mir-203a	26198104
CID 5757	hsa-mir-181a-1	unconfirmed	CID 5757	hsa-mir-34a	24050776
CID 5757	hsa-mir-124-2	26198104	CID 5757	hsa-mir-20a	21914226
CID 5757	hsa-mir-124-3	26198104	CID 5757	hsa-mir-335	unconfirmed
CID 5757	hsa-mir-346	unconfirmed	CID 5757	hsa-mir-196a-1	unconfirmed
CID 5757	hsa-mir-22	24715036	CID 5757	hsa-mir-663a	26198104
CID 5757	hsa-mir-194-2	unconfirmed	CID 5757	hsa-mir-130b	unconfirmed
CID 5757	hsa-mir-16-1	unconfirmed	CID 5757	hsa-mir-92a-1	unconfirmed
CID 5757	hsa-mir-27b	26198104	CID 5757	hsa-mir-370	unconfirmed
CID 5757	hsa-mir-27a	26198104	CID 5757	hsa-mir-373	unconfirmed
CID 5757	hsa-mir-320a	27965096	CID 5757	hsa-mir-25	unconfirmed
CID 5757	hsa-mir-26a-1	unconfirmed	CID 5757	hsa-mir-106b	28422740
CID 5757	hsa-mir-15b	26198104	CID 5757	hsa-mir-152	unconfirmed
CID 5757	hsa-mir-221	21057537	CID 5757	hsa-mir-15a	unconfirmed
CID 5757	hsa-mir-126	26198104	CID 5757	hsa-mir-18a	24245576
CID 5757	hsa-mir-16-2	–	CID 5757	hsa-mir-150	–
CID 5757	hsa-mir-29a	22334722	CID 5757	hsa-mir-9-1	26198104
CID 5757	hsa-mir-26a-2	unconfirmed	CID 5757	hsa-mir-9-2	26198104
CID 5757	hsa-mir-24-2	27030099	CID 5757	hsa-mir-196a-2	29331043

The top 1–25 miRNAs are shown in the second column while the top 26–50 are in the fifth column. As a result, 6 and 24 of the top 10 and top 50 were confirmed by the known experimental literature, respectively.

### 5-Aza-CdR

The ligand 5-Aza-CdR is an inhibitor of DNA methyltransferase (DNMT). It can reverse methylation and reactivate the expression of silenced genes.^62^ 5-Aza-CdR was able to suppress the growth of various tumors.^63^, ^64^, ^65^ We performed HSSMMA on 5-Aza-CdR and got the total ranking of all 5-Aza-CdR-associated miRNAs. The results showed that 5 of the first 10 miRNAs were confirmed by the database of SM2miR^31^ and 1 was confirmed by previous literature.^41^ Furthermore, 17 of the top 50 miRNAs were verified by SM2miR and 9 were confirmed by experiments (see Table 4). For instance, Xu et al.^66^ found that the expression level of let-7b was significantly reduced in acute lymphoblastic leukemia (ALL) patients. The 5-Aza-CdR could increase the expression of let-7b and inhibit the growth of ALL cells.^66^ Through bisulfite pyrosequencing of bladder cancer (BCa) cell lines treated with 5-Aza-CdR and 4-phenylbutyric acid (PBA), Shimizu et al.^67^ identified upregulated miRNAs by 5-aza-dC plus PBA. Among them, miR-124-2 and miR-124-3 were frequently and tumor-specifically methylated in primary cancers.^67^ In breast cancer, Manavalan et al.^68^ found that 5-Aza-CdR in combination with histone deacetylase inhibitor trichostatin A (TSA) could increase miR-200c in LY2 cells. In GBM, Ghasemi et al.^69^ revealed that treatment of U87MG cells with 5-Aza-CdR can reverse the hypermethylation status of miR-149 and increase its expression, thus decreasing target mRNA and protein levels.

**Table 4:** Top 50 miRNAs Associated with 5-Aza-2′-Deoxycytidine Were Predicted by HSSMMA Based on Dataset 1

SM	miRNA	Evidence	SM	miRNA	Evidence
CID 451668	hsa-mir-125b-1	26198104	CID 451668	hsa-mir-16-1	26198104
CID 451668	hsa-mir-125b-2	26198104	CID 451668	hsa-mir-26a-1	unconfirmed
CID 451668	hsa-mir-19b-1	unconfirmed	CID 451668	hsa-mir-181b-1	unconfirmed
CID 451668	hsa-mir-24-1	26198104	CID 451668	hsa-mir-155	26198104
CID 451668	hsa-mir-18a	unconfirmed	CID 451668	hsa-mir-132	unconfirmed
CID 451668	hsa-mir-17	26198104	CID 451668	hsa-mir-181b-2	unconfirmed
CID 451668	hsa-mir-324	unconfirmed	CID 451668	hsa-mir-194-1	unconfirmed
CID 451668	hsa-mir-203a	26577858	CID 451668	hsa-let-7c	unconfirmed
CID 451668	hsa-mir-145	26198104	CID 451668	hsa-mir-346	unconfirmed
CID 451668	hsa-mir-21	26198104	CID 451668	hsa-mir-320a	26198104
CID 451668	hsa-mir-20a	26198104	CID 451668	hsa-mir-197	unconfirmed
CID 451668	hsa-mir-19a	26198104	CID 451668	hsa-mir-199a-2	unconfirmed
CID 451668	hsa-mir-23a	unconfirmed	CID 451668	hsa-mir-200c	23626803
CID 451668	hsa-mir-27a	26198104	CID 451668	hsa-mir-128-2	unconfirmed
CID 451668	hsa-let-7a-1	unconfirmed	CID 451668	hsa-let-7a-2	unconfirmed
CID 451668	hsa-mir-181a-1	26198104	CID 451668	hsa-let-7a-3	26227220
CID 451668	hsa-mir-124-1	unconfirmed	CID 451668	hsa-mir-126	26198104
CID 451668	hsa-mir-27b	26198104	CID 451668	hsa-mir-128-1	27705931
CID 451668	hsa-let-7b	26708866	CID 451668	hsa-mir-221	unconfirmed
CID 451668	hsa-mir-328	unconfirmed	CID 451668	hsa-mir-133a-1	unconfirmed
CID 451668	hsa-mir-124-2	23200812	CID 451668	hsa-mir-342	unconfirmed
CID 451668	hsa-mir-124-3	23200812	CID 451668	hsa-mir-205	unconfirmed
CID 451668	hsa-let-7d	26802971	CID 451668	hsa-mir-149	27783537
CID 451668	hsa-mir-181a-2	26198104	CID 451668	hsa-mir-1-2	unconfirmed
CID 451668	hsa-mir-24-2	26198104	CID 451668	hsa-mir-137	26198104

The top 1–25 miRNAs are shown in the second column while the top 26–50 are in the fifth column. As a result, 6 and 24 of the top 10 and top 50 were confirmed by the known experimental literature, respectively.

## Discussion

miRNAs as a new potential therapeutic target have attracted wide attention.^70^ Moreover, large quantities of studies have certified that SMs could modulate the expression of miRNAs and, thus, have potential for treating diseases.^28^ Not surprisingly, the identification of potential miRNA-SM associations has important sense for disease therapy and drug clinical applications. In this study, we introduced a computational model of HSSMMA to predict potential miRNA-SM associations by implementing a path-constrained measurement method of HeteSim on a heterogeneous network that was established with known miRNA-SM associations, miRNA-miRNA similarity, and SM-SM similarity. The results of cross validation and case studies showed that the model could effectively predict potential miRNA-SM associations.

The effectiveness of HSSMMA is probably traceable in part to the following several factors. First, the dataset of known miRNA-SM associations used in this paper was collected from a highly reliable SM2miR database. Several other reliable datasets were also used in the model, such as SM side effect similarity, SM chemical structure similarity, gene functional consistency-based similarity for SMs and miRNAs, and disease phenotype-based similarity for miRNAs and SMs, which can greatly increase algorithm efficiency. Second, HSSMMA is a path-based relevance measure, which can effectively capture the subtle semantics information of search paths. Therefore, through selecting search paths from an SM to a miRNA in the established heterogeneous network, HSSMMA can be a useful prediction tool for prioritizing potential miRNA-SM associations. More importantly, compared with some machine-learning-based models that randomly select negative samples as training data, HSSMMA only requires positive samples as training data. The random selection of negative samples in machine-learning-based models would affect their prediction accuracy. Consequently, the prediction accuracy of the HSSMMA model is more convincing than that of the prediction models with negative samples as training data.

However, there are also some weaknesses in our proposed model: the 664 experimentally confirmed miRNA-SM associations used in this paper are far from enough. More known miRNA-SM associations need to be verified by experiment, which would contribute to the improvement of prediction accuracy for the model. Furthermore, the function for the combination of several different HeteSim scores based on search paths in our approach is relatively simple, and it could be reconstructed based on advanced machine-learning methods. Compared with previous models, HSSMMA significantly improved prediction ability for the identification of potential miRNA-SM associations. However, the prediction accuracy of HSSMMA is still not satisfactory. These factors all motivate researchers to develop more effective computational models to predict potential miRNA-SM associations based on the reliable biological datasets.

## Materials and Methods

### SM-miRNA Associations

The dataset of 664 experimentally verified miRNA-SM associations used in this study was downloaded from the SM2miR database.^40^ Dataset 1 includes 541 miRNAs, 831 SMs, and 664 known miRNA-SM associations, of which only a portion of miRNAs and SMs participate in the known miRNA-SM associations. Dataset 2 includes 39 SMs, 286 miRNAs, and 664 known miRNA-SM associations, where all SMs and miRNAs participate in the known associations. All the known SM-miRNA associations are listed in Table S2. More importantly, we constructed an adjacency matrix *A* to indicate the known miRNA-SM associations. If SM *i* is associated with miRNA *j*, the entity *A*(*i,j*) is 1, otherwise 0. We further used *ns* and *nm* to indicate the number of SMs and miRNAs in the dataset.

### Integrated SM Similarity

Lv et al.^31^ calculated an SM similarity score based on SM side effect similarity $S_{S}^{s}$,^71^ gene functional consistency-based similarity for SMs $S_{S}^{T}$,^72^ SM chemical structure similarity $S_{S}^{C}$,^73^ and disease phenotype-based similarity for SMs $S_{S}^{s}$.^71^ Here, SM side effect similarity was computed by using the Jaccard score^71^ based on the dataset of SM drug side effects that was collected from SIDe Effect Resource (SIDER).^74^ Gene functional consistency-based similarity for SMs was reflected by employing the Gene Set Functional Similarity (GSFS) method^72^ on the dataset of target genes of the SMs obtained from DrugBank^75^ and Therapeutic Targets Database (TTD).^76^ Moreover, SM chemical structure similarity was gained by enforcing a graph-based method of SIMilar COMPound (SIMCOMP),^73^ in light of the dataset of SM chemical structure extracted from the drug and compound sections of the Kyoto Encyclopedia of Genes and Genomes (KEGG) ligand database.^77^ At last, based on the dataset of SM-related diseases downloaded from DrugBank and TTD, disease phenotype-based similarity for SMs was defined by employing Jaccard score.

To balance the four types of SM similarity and reduce the deviation of each similarity, the integrated SM similarity $S_{S}$ can be defined as follows:

(Equation 1) $$S_{S}=\;\left(\beta_{1}S_{S}^{D}+\beta_{2}S_{S}^{T}+\beta_{3}S_{S}^{C}+\beta_{4}S_{S}^{s}\right)/\sum_{j}\beta_{j}\left(j=1,2,3,4\right)\;\;,$$

where the default value $\beta_{j}=1$ means that the separated similarities have the same weight. In this paper, we have released the integrated similarity of all 831 SMs that were used in dataset 1 (see Table S3).

### Integrated miRNA Similarity

In the same way, Lv et al.^31^ also calculated miRNA similarity by a weighed combination of gene functional consistency-based similarity for miRNAs $S_{M}^{T}$and disease phenotype-based similarity for miRNAs $S_{M}^{D}$.^71^, ^72^ On the basis of the target genes of each miRNA from TargetScan,^78^ gene functional consistency-based similarity for miRNAs could be gained through carrying out the GSFS method^72^ to compute functional consistency of their miRNA target gene sets;^72^ disease phenotype-based similarity for miRNAs was calculated by using Jaccard score according to the dataset of miRNA-related diseases extracted from Human MicroRNA Disease Database (HMDD) version (v.)2.0^79^ database and miR2Disease^80^ and PhenomiR^81^ databases. At last, the integrated miRNA similarity $S_{M}$ can be defined as follows:

(Equation 2) $$S_{M}=\left(\alpha_{1}S_{M}^{D}+\alpha_{2}S_{M}^{T}\right)/\sum_{i}\alpha_{i}\left(i=1,2\right),$$

where the default value $\alpha_{i}=1$ means the separated similarities possess the same weight. Likewise, we have released the integrated similarity of all 541 miRNAs that were used in dataset 1 (see Table S4).

### HSSMMA

**Figure 4:**
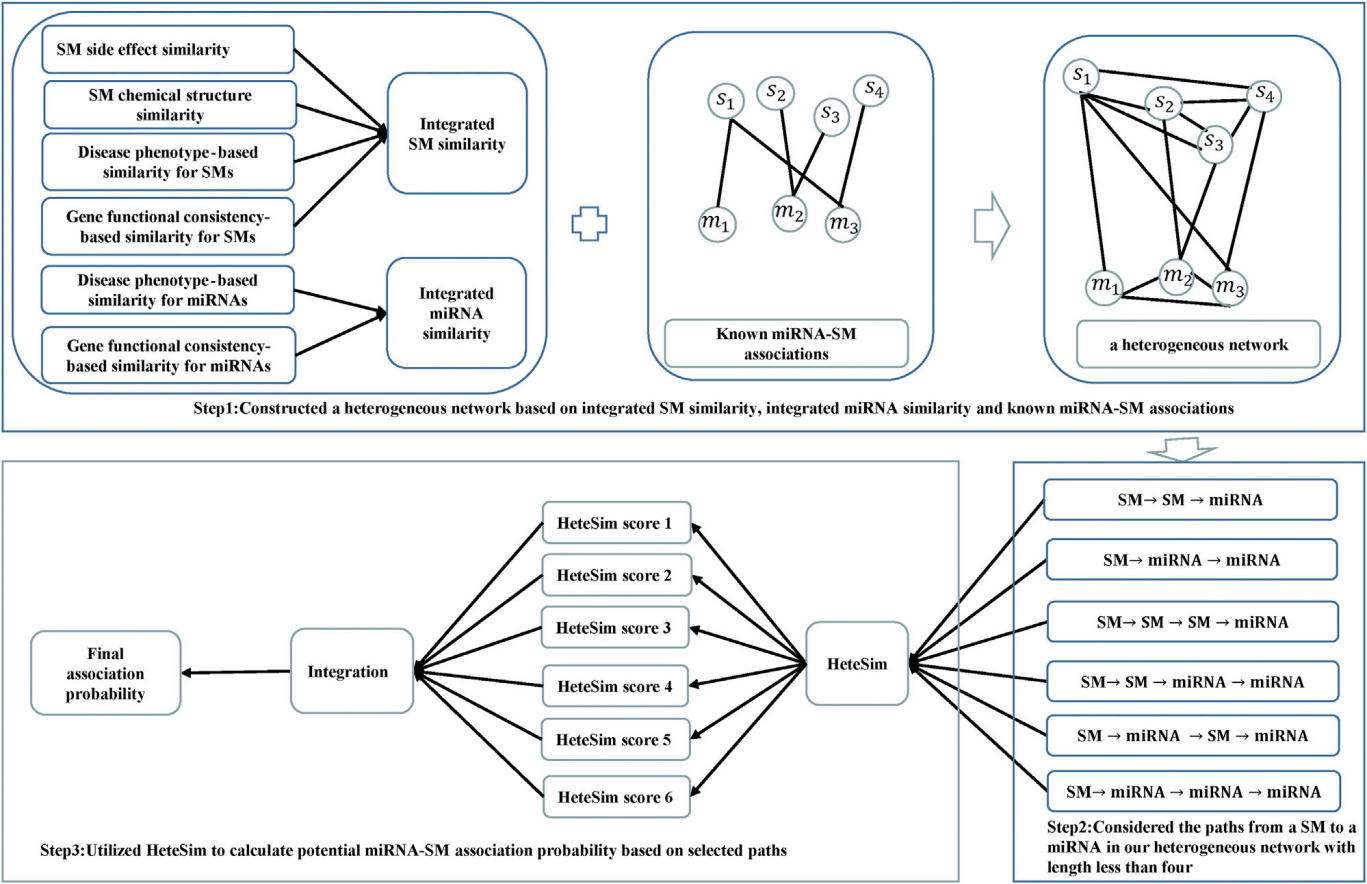
Flowchart of Potential miRNA-SM Association Prediction Based on HSSMMA We constructed a miRNA-SM heterogeneous network based on known miRNA-SM associations, integrated SM similarity, and integrated miRNA similarity, and we obtained potential association probability of miRNA-SM associations by implementing HeteSim on the heterogeneous.

In this study, motivated by previous research introduced by Zheng et al.,^82^ we combined integrated SM similarity, integrated miRNA similarity, and known miRNA-SM associations into a heterogeneous network (see Figure 4 step 1), and we proposed a computational method of HSSMMA to infer potential associations between miRNAs and SMs by implementing HeteSim on an established heterogeneous network. Here, HeteSim is a path-based measurement method that evaluates the relatedness of object pairs depending on the considered search paths that connect two objects through a sequence of node types.^39^ Unlike homogeneous networks, the paths in heterogeneous networks possess semantics. Consequently, on the basis of a basic idea that similar objects are relevant to similar objects, Kong and colleagues^39^ designed the uniform and symmetric measure of HeteSim for arbitrary paths to compute the associations of heterogeneous objects. For a given path (symmetric or asymmetric), the measure can calculate the association score of each heterogeneous object pair. Moreover, HeteSim has been implemented in the identification of lncRNA-protein interactions,^82^ disease-gene associations,^83^ and lncRNA-protein associations,^84^ which demonstrated the high precision and good performance of HeteSim. Therefore, we carried out HeteSim measurement to infer potential miRNA-SM associations.

SM and miRNA are two types of objects in the miRNA-SM heterogeneous network. $SM\overset{R}{\to }miRNA$ indicates a relationship from type SM to type miRNA and the length *l* of the path is 1, where SM and miRNA represent the source type and target type of relationship *R*, respectively. A is an adjacent matrix between SM and miRNA, and the row-normalized matrix of A can be defined as follows:

(Equation 3) $$T_{sm}\left(i,j\right)=\frac{A\left(i,j\right)}{\sum_{k=1}^{nm}A\left(i,k\right)},$$

where $T_{sm}$ can be regarded as the transition probability matrix from SM to miRNA. In the same way, $T_{ss}$ and $T_{mm}$ can be calculated to indicate the transition probability matrix from SM to SM and miRNA to miRNA by obtaining the row-normalized matrix of integrated SM similarity $S_{S}$ and integrated miRNA similarity $S_{M}$, respectively.

#### Definition 1 (Reachable Probability Matrix)

A reachable probability matrix for path $P=\left(A_{1},A_{2},\cdots ,A_{l+1}\right)$ in a heterogeneous network can be defined as follows:

(Equation 4) $$R_{p}=T_{A_{1}A_{2}}T_{A_{2}A_{3}}\cdots T_{A_{l}A_{l+1}}.$$

Given two entities, a∈SM and b∈miRNA, the relatedness between *a* and *b* can be calculated based on the HeteSim measure. Because HeteSim is a path-based relevance measurement, it is significant to consider different search paths that connect SM and miRNA. In general, the contribution of short paths may be more significant than long paths. Therefore, we only considered the paths from an SM to a miRNA in our heterogeneous network with length less than four (see Figure 4 step 2). If the length *l* of the search path is even, the search path can be divided into two parts with equal length. As we can see from Figure 4 step 2, a relevance path from an SM to a miRNA along a sequence of object types with length two can be indicated as $SM\overset{R_{1}}{\to }SM\overset{R_{2}}{\to }miRNA$. The search path *P* = (*SM, SM, miRNA*) can be expressed as $P=\left(P_{L}P_{R}\right)$, where the left path $P_{L}=SM\overset{R_{1}}{\to }SM$ and the right path $P_{R}=SM\overset{R_{2}}{\to }miRNA$. The reachable probability matrix for the left and right paths in the heterogeneous network are $R_{P_{L}}$ = $T_{ss}$ and $R_{P_{R}}=T_{sm}$, respectively. Finally, the HeteSim score between *a* and *b* based on the path *P* can be calculated as follows:

(Equation 5) $$HeteSim\left(a,b|P\right)=\frac{R_{P_{L}}\left(a,:\right)\times {\left(R_{P_{R}^{-1}}\left(b,:\right)\right)}^{T}}{R_{P_{L}}{\left(a,:\right)}_{2}\times R_{P_{R}^{-1}}{\left(b,:\right)}_{2}},$$

where $P_{R}^{-1}$ is the reverse path of $P_{R}$. We give an example of using HeteSim to calculate the score between $s_{3}$ and $m_{1}$ under the path *P* = (*SM, SM, miRNA*), i.e., $R_{P_{L}}=T_{ss}$ and $R_{P_{R}^{-1}}=T_{ms}$:

(Equation 6) $$HeteSim\left(s_{3},m_{1}|P\right)=\frac{T_{ss}\left(3,:\right)\times {\left(T_{ms}\left(1,:\right)\right)}^{T}}{T_{ss}{\left(3,:\right)}_{2}\times T_{ms}{\left(1,:\right)}_{2}}.$$

For the other three search paths with the length two listed in Figure 4 step 2, the corresponding HeteSim scores can also be calculated. If the length *l* of the search path is odd, the path cannot be equally divided into two parts. For example, a relevance path from an SM to a miRNA along a sequence of object types with length three can be indicated as $SM\overset{R_{1}}{\to }SM\overset{R_{2}}{\to }SM\overset{R_{3}}{\to }miRNA$. Therefore, we consider the following two cases: (1) $P_{L}=SM\overset{R_{1}}{\to }SM\overset{R_{2}}{\to }SM$ and $P_{R}=SM\overset{R_{3}}{\to }miRNA$. The reachable probability matrix for the left and right paths can be defined as $R_{P_{L}}$ = $T_{ss}T_{ss}$ and $R_{P_{R}}=T_{sm}$, respectively; (2) $P_{L}=SM\overset{R_{1}}{\to }SM$ and $P_{R}=SM\overset{R_{2}}{\to }SM\overset{R_{3}}{\to }miRNA.$ The reachable probability matrix of the left and right paths are $R_{P_{L}}$ = $T_{ss}$ and $R_{P_{R}}=T_{ss}T_{sm}$, respectively. The final HeteSim value of the search path *P* = (*SM, SM, SM, miRNA*) would be obtained through calculating the average of two HeteSim values based on the above two different cases. We can also calculate HeteSim score of another search path with the length three listed in Figure 4 step 2.

Consequently, we would obtain six different HeteSim scores of six relevance paths and integrate these scores to obtain the final association scores between *a* and *b*, which can be defined as follows (see Figure 4 step 3):

(Equation 7) $$S\left(a,b\right)=\sum_{l=2}^{3}\left(\beta^{l-1}\times \sum_{P_{i}\in \psi_{l}}HeteSim\left(a,b|P_{i}\right)\right),$$

where *a* is the entity of object type SM, *b* is the entity of object type miRNA, and $\psi_{l}$ is the set of path $P_{i}$from SM to miRNA with the length of *l*. β is a decay factor, which can further punish the longer paths.

## Author Contributions

All authors contributed important elements to the work presented herein. J.Q. implemented the experiments, analyzed the results, and wrote the paper. X.C. conceived the project, developed the prediction method, designed the experiments, analyzed the results, revised the paper, and supervised the project. Y.-Z.S., Y.Z., S.-B.C., Z.M., Z.-H.Y., and J.-Q.L. analyzed the results and revised the paper. All authors read and approved the final manuscript.

## Conflicts of Interest

The authors declare no competing interests.

## Supplemental Information

Document S1. Table S1 We applied HSSMMA to prioritize all the candidate miRNA-SM pairs based on all the known miRNA-SM associations in SM2miR database as training samples. This prediction result is released for further experimental validation and research.

Table S2. The Dataset of 664 Known miRNA-SM Associations between 541 miRNAs and 831 SMs

Table S3. The Integrated Similarity of All 831 SMs Used in Dataset 1

Table S4. The Integrated Similarity of All 541 miRNAs Used in Dataset 1
